# Severe Sepsis Caused by California Serogroup Orthobunyavirus

**DOI:** 10.3201/eid2110.150394

**Published:** 2015-10

**Authors:** Daniel K. Rogstad, Elizabeth Schiffman, David Neitzel, Larry M. Baddour

**Affiliations:** Loma Linda University, Loma Linda, California, USA (D.K. Rogstad);; Mayo Clinic, Rochester, Minnesota, USA (D.K. Rogstad, L.M. Baddour);; Minnesota Department of Health, St. Paul, Minnesota, USA (E. Schiffman, D. Neitzel)

**Keywords:** California encephalitis virus, arbovirus infections, orthobunyavirus, sepsis, Minnesota, viruses, California serogroup, United States

**To the Editor:** The California serogroup (CAL) of orthobunyaviruses, including Jamestown Canyon virus (JCV) and La Crosse virus (LACV), can cause neuroinvasive disease in humans (*1*–*3*). The epidemiology and clinical spectrum of LACV are well described (*3*), whereas less is known about JCV. 

JCV is distributed throughout temperate North America, and its transmission cycle involves ungulates and several mosquito genera (*4*–*6*). The first human case of JCV disease in Minnesota, USA, was reported in 2013 (*7*), although serologic evidence of the virus had been previously documented in the white-tailed deer population (*8*). Of the 22 human cases reported to the Centers for Disease Control and Prevention in the United States during 2013, 15 were neuroinvasive (*7*). However, given the lack of widespread testing availability and the few reported cases, the actual clinical spectrum of JCV infection, unlike that of LACV, remains unclear. We report a case of severe sepsis in an adult with antibodies against CAL and suspected JCV disease.

A 62-year-old man from Waseca County, Minnesota, who had type 2 diabetes mellitus, obesity, migraine headaches, depression, and anxiety, had been vacationing for 1 week at his cabin in Carlton County, Minnesota. On July 9, 2014, he experienced diffuse myalgia, arthralgia, and headache. He sought treatment July 10 at a local urgent care facility for fever, rigors, sweats, weakness, dizziness, poor appetite, and mild shortness of breath, but he denied having cough, chest pain, gastrointestinal symptoms, neck stiffness, and photophobia. He described extensive mosquito exposure the prior week. On physical examination, temperature was 38.8°C, and he had multiple skin lesions consistent with mosquito bites. A 5-cm, lacy, petechial skin rash was observed on the right inner thigh. Chronic venous stasis changes of the lower extremities were noted, and a faint systolic cardiac murmur was heard. Peripheral leukocyte count was 11.2 × 10^9^ cells/L, and electrolyte levels were within reference ranges. Serologic screenings for likely infectious agents were negative (Table). Empiric doxycycline was initiated.

**Table:** Pathogen testing in patient with undifferentiated severe sepsis, Minnesota, USA, 2014*

Pathogen tested	Serum sample	
	Acute phase	Convalescent phase
HIV-1/-2	Ag/Ab screen negative	
*Leptospira*	IgM/IgG negative	
*Cryptococcus*	Antigen screen negative	
*Anaplasma phagocytophilum*	IgG <1:64, PCR negative	
*Ehrlichia chaffeensis*	IgG <1:64, PCR negative	
*Ehrlichia ewingii/canis*	PCR negative	
*Ehrlichia muris*–like	PCR negative	
*Borrelia burgdorferi*	Ab screen negative	Ab screen negative
*Babesia microti*	IgG <1:64, PCR negative	
*Babesia duncani*	PCR negative	
*Babesia divergens* strain MO-1	PCR negative	
West Nile virus	IgM/IgG and PCR negative	IgM/IgG negative
Eastern equine encephalitis virus	IgM/IgG <1:10	IgM/IgG <1:10
Western equine encephalitis virus	IgM/IgG <1:10	IgM/IgG <1:10
St. Louis encephalitis virus	IgM/IgG <1:10	IgM/IgG <1:10
California serogroup virus	IgM ≥1:10,† IgG 1:10	IgM 1:80, IgG 1:320
Powassan/tick-borne encephalitis virus‡	IgM negative	
La Crosse virus‡	IgM negative, PRNT <10	PRNT 320
Jamestown Canyon virus‡	IgM negative, PRNT 160	PRNT 10,240

*Positive control value for PRNT testing was >1,280. Ag, antigen; Ab, antibody, Ig, immunoglobulin, PRNT, plaque reduction neutralization testing. †Additional dilution of result not performed. ‡Testing performed at the Arboviral Diseases Branch Laboratory, Centers for Disease Control and Prevention, Fort Collins, Colorado, USA.

Two days later, he experienced worsening myalgia, arthralgia, fevers, profuse sweating, nausea, and vomiting and visited a local emergency department. Mild confusion was noted on examination. Temperature was 37.9°C, and blood pressure was 69/32 mm Hg, with minimal improvement despite intravenous fluid administration. Peripheral leukocyte count was 16.2 × 10^9^ cells/L, serum creatinine and blood-urea nitrogen levels were elevated at 1.7 and 37 mg/dL, respectively, and liver enzyme and lactate levels were normal. Results of a chest radiograph were unremarkable. Dopamine and broad-spectrum antibiotic drugs were initiated, and he was transferred to Mayo Clinic (Rochester, Minnesota) in the intensive care unit. At that time, no confusion was noted, nor signs or symptoms of neuroinvasion that would have warranted cerebrospinal fluid examination or neuroimaging. 

Hypotension resolved overnight, and leukocytosis and serum creatinine levels improved. A low-grade fever of 38.0°C was documented in the evening of July 14. Because the overall etiology of the illness remained unclear, a computed tomography scan of the abdomen and pelvis was performed to investigate potential intraabdominal sources of sepsis, but results were unrevealing. By hospital day 5, blood cultures remained negative, leukocytosis resolved, and creatinine levels normalized. Antibiotic drugs were discontinued, and the patient was discharged with a 2-week course of oral doxycycline. Soon after discharge, an acute-phase serum specimen was positive for a CAL orthobunyavirus (Table).

During the next 3 weeks, the man sought treatment multiple times as an outpatient for diarrhea, lightheadedness, dizziness, headache, fatigue, difficulties concentrating, and subjective memory loss. These symptoms gradually resolved over 2 months. Convalescent-phase serum samples were drawn 3 weeks after dismissal and showed 32-fold increased antibody titers against CAL. The Minnesota Department of Health was notified, and plaque reduction neutralization testing was ordered to determine the specific virus. Increases in the neutralizing antibody titers against JCV and LACV were noted; although the absolute titer was greater for JCV (Table), a final determination of which virus caused the patient’s illness could not be made. Neutralizing antibodies in the acute-phase specimen in the absence of IgM suggests that the patient was previously exposed to an orthobunyavirus. 

Antibody cross-reactivity between closely related arboviruses such as LACV and JCV is not unusual, and similar antigenic sin has been described with other orthobunyaviruses (*9*). Epidemiologic factors, including time of year, location of mosquito exposure, and the predilection of LACV disease for children, point to JCV infection in this case, although it cannot be confirmed.

Previous reports of JCV disease have focused on the ability of this virus to cause asymptomatic infection, nonspecific mild febrile illness, or severe neuroinvasive disease (*4*,*7*,*10*). This case illustrates a suspected JCV infection causing undifferentiated severe sepsis, which has not, to our knowledge, been previously reported. Initial suspicion for acute neuroinvasive disease was low, and neurologic imaging and cerebrospinal fluid sampling were not performed. We recommend that testing for CAL (and specifically for JCV) infection should be strongly considered in the setting of severe sepsis in adults with substantial exposure to mosquitoes and no other identifiable source of sepsis.
